# Richmond Academy of Medicine and Surgery

**Published:** 1899-03

**Authors:** 


### Society Notes.

### Richmond Academy of Medicine and Surgery.

MEETING HELD JANUARY 24, 1899.

Dr. E. C. Levy, President, in the chair; Dr. Mark W. Peyser, Secretary and Reporter.

Dr. A. M. Phelps, of New York, read a paper* on "Lateral Curvature of the Spine and Pott's Disease."

Dr. Stuart McGuire said that he had listened with interest and profit to Dr. Phelps' admirable discussion; that the subject of Pott's disease was one of peculiar interest to him, as he had been the victim of the disease during childhood; that he had been a patient of Dr. Lewis Sayre; that he had been the subject of many experiments, and that he believed he was the original case upon whom the plaster of Paris jacket was applied; that although twenty-five years had elapsed he could remember how Dr. Sayre placed him face downward across his knees and by separating his

[*Printed in our February number. -ED.]

legs and producing extension thus relieved pain and reduced deformity. This was the inception of a principle now carried out by suspension. That he remembered how Dr. Sayre placed his broad hands on either side of his spinal column and, by gentle pressure, maintained the correction secured and gave support and immobilization to the back. This was the inception of the principle now carried out by the plaster cast. Dr. Mcguire said that the first attempt at the practical application of the brace consisted in laying him upon a table and producing extension by manual traction on his head and feet and then the application of alternate layers of squares of flannel and wet plaster to his back. This formed a "turtle shell," which was held in place by circular turns of a cotton bandage. Dr. McGuire then outlined the evolution of the plaster jacket, and spoke of its advantages—cheapness and effectiveness, and its disadvantages—short life and lack of cleanliness.

In regard to the aluminum corset invented by Dr. Phelps he said it was a perfect substitute for the plaster brace, combining all of its virtues and having none of its vices; that unfortunately, owing to its cost, it would never be widely adopted, but for the well-to-do it was a luxury which should not be lost sight of.

In conclusion, Dr. McGuire spoke of the muscular atrophy and diminished chest expansion which resulted from the long use of any brace, and of the advisability of taking them off as soon as they could be safely discarded. He asked Dr. Phelps what were the evidences of cure of Pott's disease and what was his rule as to the length of time a brace should be worn.

Dr. J. A. Hodges said he would be glad if Dr. Phelps told the ultimate results of lateral curvatures and Pott's disease on respiration, and also the forms of paralysis in patients left untreated. He was surprised that there was not more paralysis resulting from destruction of the vertebrae and from pressure on and degeneration of the spinal nerves.

Dr. George Ross reported the following case: A theological student went coasting the hillside and caught cold. He was unconscious of having sustained an injury, and yet in a few days he found himself unable to walk up the steps. His feet were leaden. He was placed in the hospital of the school, where he remained for six weeks. No improvement following the treatment advised, he was sent to a hospital in Baltimore. Paraplegia with myelitis was diagnosed, and a fatal prognosis made. Two months of observation failed to warrant a change of opinion, and the patient was sent home to die. Being the family physician, Dr. Ross was summoned to see the patient, and found him with thighs flexed on the abdomen, knees close under his chin, limbs in spastic rigidity, emunctions paralyzed and pains excrutiating. The history furnished seemed to warrant the conclusion that the case was one of acute ascending myelitis, with paralysis from pressure. Months rolled by without material change other than the advent of girdle pains of the abdomen and chest and harassing bronchial cough, with difficult asthmatic breathing and repeated threatenings of impending suffocation. Then there appeared a swelling near the cervico-dorsal vertebral junction and a culminating abscess, which was lanced. It was long in healing, and, though naturally to be looked for, there is no record of necrosing bone escaping from its cavity. The presence of this abscess proved clearly to his mind that the case was one of Pott's disease of the upper dorsal vertebrae. No mechanical appliance was at any time used, and the reliance for treatment rested solely on spinal counter irritants, constitutional reconstructives and supportives and an intelligent dietary. The surprising outcome of this case is that today, though deformed by a posterior upper dorsal curvature, the patient is healthy and vigorous, and, while engaged in no special work, is quite competent to do many things.

Dr. Phelps said, in closing the discussion, that the mode of manufacturing the aluminum corset was to extend the patient and apply the bandages so as to make a plaster cast. This was cut off, stuffed with oakum and plaster of Paris, after which shellac was applied to the stuffing. Sheets of the softest aluminum were laid on the mold and shaped with a wooden hammer. It was then coated inside and out with white shellac and alcohol to prevent the action of perspiration. He said he had hoped that as time progressed the apparatus could be made and sold at a lower price.

How aptly Dr. McGuire tells of Dr. Sayre! The orthopedic hand is the best brace made; it can mold the corset to fit, and is in partnership with all the ideas conducive to best results.

The indications of cure are the same as those of hip-joint disease. Here I never remove the brace until the limit of movement is increased, and so I do in Pott's disease, which is never cured in less than three years.

Atrophy is always produced by degeneration of the nervous end plates in the muscles. Braces do not produce atrophy. If a brace gives room in front there is no interference with the play of the chest.

The wire corset does not support as it should. Patients using it are two inches taller when placed in a plaster corset. The aluminum cast fits the patient like a French corset.

A complete cure cannot be produced in lateral curvature, because the ribs overlap, the intercostal muscles are shortened and the spaces obliterated. The ribs cannot be separated except by means of the knife, and if this is used the patient dies.

Concerning paralysis, I will venture to say that from 15 to 20 per cent. of patients afflicted with Pott's disease manifest it at some stage, it varying from involvement of groups of muscles to total paralysis. Of the estimated 20 per cent., 95 per cent. will recover without operation from the complication; the remainder will not. It is not always due to bending; sometimes it is from involvement of the canal, producing thickening and pressure myelitis. In some cases I have seen tubercular meningitis; in others penetration of an abscess. My observation is that those cases attended by bladder and rectal incontinence never recover, but I have seen recovery where these organs were only irritated.

Dr. Ross' case was one of osteo-myelitis recovering without treatment, but this should not be an argument against treatment.

